# Kidney cancer in the Middle East and North Africa region: a 30-year analysis (1990–2019)

**DOI:** 10.1038/s41598-024-64521-7

**Published:** 2024-06-14

**Authors:** Saeid Safiri, Kamaleddin Hassanzadeh, Amir Ghaffari Jolfayi, Seyed Ehsan Mousavi, Kimia Motlagh Asghari, Seyed Aria Nejadghaderi, Nima Naghdi-Sedeh, Maryam Noori, Mark J. M. Sullman, Gary S. Collins, Ali-Asghar Kolahi

**Affiliations:** 1 Social Determinants of Health Research Center, Department of Community Medicine, Faculty of Medicine, Tabriz University of Medical Sciences, Tabriz, Iran; 2 Clinical Research Development Unit of Tabriz Valiasr Hospital, Tabriz University of Medical Sciences, Tabriz, Iran; 3 Department of Urology, Faculty of Medicine, Tabriz University of Medical Sciences, Tabriz, Iran; 4 Student Research Committee, School of Medicine, Shahid Beheshti University of Medical Sciences, Tehran, Iran; 5 Neurosciences Research Center, Aging Research Institute, Tabriz University of Medical Sciences, Tabriz, Iran; 6 Hematology and Oncology Research Center, Tabriz University of Medical Sciences, Tabriz, Iran; 7 HIV/STI Surveillance Research Center, and WHO Collaborating Center for HIV Surveillance, Institute for Futures Studies in Health, Kerman University of Medical Sciences, Kerman, Iran; 8 Systematic Review and Meta-Analysis Expert Group (SRMEG), Universal Scientific Education and Research Network (USERN), Tehran, Iran; 9 Urology Research Center, Tehran University of Medical Sciences, Tehran, Iran; 10 Department of Life and Health Sciences, University of Nicosia, Nicosia, Cyprus; 11 Department of Social Sciences, University of Nicosia, Nicosia, Cyprus; 12 Centre for Statistics in Medicine, NDORMS, Botnar Research Centre, University of Oxford, Oxford, UK; 13 NIHR Oxford Biomedical Research Centre, Oxford University Hospitals NHS Foundation Trust, Oxford, UK; 14 Social Determinants of Health Research Center, Shahid Beheshti University of Medical Sciences, Tehran, Iran

**Keywords:** Kidney neoplasms, Incidence, Years lived with disability, Epidemiology, MENA, Cancer epidemiology, Epidemiology

## Abstract

Kidney cancer, a type of urogenital cancer, imposes a high burden on patients. Despite this, no recent research has evaluated the burden of this type of cancer in the Middle East and North Africa (MENA) region. This study explored the burden of kidney cancer from 1990 to 2019 according to age, sex and socio-demographic index (SDI). The Global Burden of Disease (GBD) 2019 data was utilized to estimate the incidence, death, and disability-adjusted life-years (DALYs) caused by kidney cancer. These estimates were reported as counts and as age-standardised rates with 95% uncertainty intervals (UIs). The estimated age-standardised incidence, mortality, and DALY rates of kidney cancer in 2019 were 3.2 (2.8–3.6), 1.4 (1.2–1.6), and 37.2 (32.0–42.6) per 100,000, respectively. Over the period from 1990 to 2019, these rates have increased by 98.0%, 48.9%, and 37.7%, respectively. In 2019, the United Arab Emirates, Qatar, and Lebanon had the largest age-standardised incidence, mortality, and DALY rates. The smallest age-standardised incidence rates were seen in Yemen, Afghanistan, and the Syrian Arab Republic. Additionally, the smallest age-standardised mortality and DALY rates were observed in the Syrian Arab Republic, Yemen, and Morocco. The highest incidence rates were found among individuals aged 75–79 in both males and females. In 2019, the MENA/Global DALY ratio exceeded one for females aged 5–19 age and males aged 5–14, compared to 1990age groups in males. The burden of kidney cancer consistently rose with increasing SDI levels from 1990 to 2019. The increasing burden of kidney cancer highlights the urgent need for interventions aimed at improving early diagnosis and treatment in the region.

## Introduction

On a global scale, the number of newly diagnosed cancer cases rose from 18.7 million in 2010 to 23.6 million in 2019, representing a rise of about 26%. During the same period, cancer-related deaths worldwide grew from 8.3 million to 10.0 million^1^. In 2019 cancer was the second largest burden and the cause of the second highest number of deaths globally^1^. Kidney cancer is among the most prevalent cancers in the world, accounting for 2.3% of all cancers in 2020^2^ and 1.8% of all cancer-related mortality^2^. The worldwide incidence rate of kidney cancer has been increasing since the 1970s, with substantial inter-country variations^3^.

Renal cell carcinoma (RCC) stands as the most widespread type of kidney cancer. It is primarily asymptomatic and normally presents as metastatic disease at the time of diagnosis. RCC accounts for 90% of the incident cases and is responsible for most of the morbidity and mortality associated with the disease^4^. Kidney cancer imposes a significant burden, largely due to the high economic costs associated with RCC^5^. Although the causes of kidney cancer are multifactorial and not fully understood, several risk factors have been identified, including obesity, tobacco smoking, a history of hypertension, and/or chronic kidney disease^3^.

Current information on cancer mortality, incidence, and disability-adjusted life years (DALYs) are essential for informed healthcare decision-making. This data informs priority areas, the effectiveness of prevention programs, resource allocation, and whether additional research is needed. Given the large number of people with kidney cancer and its substantial impact on patients and the healthcare system, it is crucial to gain a comprehensive understanding of its current burden in the region. The MENA region, comprising 21 countries, exhibits diverse cultural and lifestyle-related variations.

The worldwide burden of kidney cancer has been documented utilising information from the Global Burden of Disease (GBD) study 2017^6^ and GBD 2019^7^. Furthermore, several studies have reported the regional incidence and mortality of kidney cancer^8,9^, but not specifically in MENA. Therefore, the current study examines the incidence, mortality, and DALYs associated with the disease in MENA, stratified by age, sex, and socio-demographic index (SDI) from 1990 to 2019. This comprehensive study aims to provide a clearer picture of the kidney cancer burden in the MENA region, which can help in tailoring specific healthcare strategies and interventions.

## Methods

### Overview

The GBD study has monitored the burden of 369 diseases and injuries worldwide since 1990^10^. The MENA region comprises 21 countries: Afghanistan, Algeria, Bahrain, Egypt, Iraq, Islamic Republic of Iran, Jordan, Kuwait, Lebanon, Libya, Morocco, Oman, Palestine, Qatar, Saudi Arabia, Sudan, Syrian Arab Republic, Tunisia, Turkey, United Arab Emirates, and Yemen. The sections below summarise the methods used by GBD 2019 to model the burden of kidney cancer, but for a detailed description please see one of the capstone publications^10,11^. In addition, the data used may be viewed here: https://vizhub.healthdata.org/gbd-compare/ and http://ghdx.healthdata.org/gbd-results-tool.

### Estimation framework

Kidney cancer was classified using the International Classification of Diseases, version 10 (ICD10), which included the following codes C64–C64.2, C64.4–C64.6, C64.8–C64.9, C65– C65.2, C65.9, D30.0–D30.1, and D41.0–D41.1^10^. The vital registration, verbal autopsy, and cancer registry data were used to obtain the cause of death database and transformed them into estimates of mortality using the mortality-to-incidence ratios^12^. The cause-of-death ensemble model (CODEm) was created utilising these mortality estimates and data sourced from the cancer registries and vital registration system^12^. CODEm was used to model mortality for all locations that had sparse or no available data, and the covariates used to model kidney cancer have been previously reported^12^. The CoDCorrect algorithm was applied to correct the aggregated number of predicted single-cause death estimates in each age-sex-state-year group. Following this, the mortality estimates were divided by the mortality-to-incidence ratio, in order to estimate the incidence of kidney cancer^12^. In order to model the 10-year prevalence of kidney cancer, the survival of each incidence cohort was modelled using the mortality-to-incidence ratio as a scalar. This approach positioned each nation on a spectrum from the theoretical best to the worst survival rates. Impairment was measured by separating the overall prevalence into the four different stages: diagnosis and primary treatment, controlled, metastatic, and terminal^12^. The diagnostic and primary therapy phase spans from the commencement of symptoms to the cessation of treatment. After the treatment has finished the controlled phase starts, which in turn finishes after one of the following occurs: cure (recurrence- and progression free survival after 10 years); death from another cause; or moving to the metastatic phase.

Table S1 shows the disability weights for the four sequelae. Any patients who survived longer than ten years were considered cured and classified into either the diagnosis and primary treatment phase or the controlled phase. The durations of the three different phases were as follows: diagnosis and primary therapy (5.3 months), metastatic phase (5.38 months), and the terminal phase (1 month)^10,12^.

### Severity and years lived with disability

Sequela-specific years lived with disability (YLDs) were modelled by multiplying the sequela-specific prevalence rates with their respective disability weights. The lay descriptions of each sequelae and their disability weights have previously been reported^10,12^. The YLD and years of life lost (YLL) were added together to estimate the DALYs attributable to kidney cancer. All estimates were standardized using the GBD standard population^10,12^ and had 95% confidence intervals (UIs). The 95% confidence intervals were produced by running 1000 draws at each stage of the modelling process, and included the 25th and 975th values of the ordered draws. The relationship SDI has with the burden of kidney cancer was investigated utilizing smoothing splines^13^. The SDI ranges from 0 (least developed) to 1 (most developed), was estimated by combining the fertility rate among those younger than 25, average number of years in education for individuals older than 15, and the mean income (smoothed over the prior decade). The figures were made using R (Version 3.5.2).

### Ethics approval and consent to participate

The present study was reviewed and approved by Ethics Committee of Shahid Beheshti University of Medical Sciences, Tehran, Iran (Ethics code: IR.SBMU.RETECH.REC.1402.046).

## Results

### The MENA region

There were 15.7 thousand (95% UI: 13.6 to 18.0) incident cases of kidney cancer in 2019, and an age-standardised incidence rate of 3.2 (2.8 to 3.6) per 100,000. This represents a 98.0% (55.5 to 157.4) increase compared to the 1990 rate (Table 1 and Table S2). Kidney cancer was responsible for 6.0 thousand (5.2 to 6.9) deaths in 2019, with an age-standardised death rate of 1.4 (1.2 to 1.6), which represents a 48.9% (16.8 to 109.1) increase compared to 1990 (Table 1 and Table S3). Additionally, there were 183.6 thousand (157.6 to 211.8) DALYs due to the disease in 2019, with an age-standardised rate of 37.2 (32.0 to 42.6) per 100,000, which represents a 37.7% (5.8 to 83.6) increase compared to 1990 (Table 1 and Table S4).

**Table 1:** Age-standardised incidence, death, and DALYs for kidney cancer in the Middle East and North Africa region for both sexes in 2019 and the percentage change in the age-standardised rates during the period 1990–2019.

	Incidence (95% UI)			Deaths (95% UI)			DALY (95% UI)		
	Counts (2019)	ASRs (2019)	Pcs in ASRs 1990–2019	Counts (2019)	ASRs (2019)	Pcs in ASRs 1990–2019	Counts (2019)	ASRs (2019)	Pcs in ASRs 1990–2019
North Africa and Middle East	15,742 (13,603 , 18,040)	3.2 (2.8 , 3.6)	98 (55.5 , 157.4)	6003 (5161 , 6891)	1.4 (1.2 , 1.6)	48.9 (16.8 , 109.1)	183,630 (157,587 , 211,815)	37.2 (32 , 42.6)	37.7 (5.8 , 83.6)
Afghanistan	316 (218 , 432)	1.5 (1 , 2.1)	33.8 (-6.7 , 92.9)	143 (94 , 205)	1 (0.6 , 1.4)	18.8 (-16.4 , 69.4)	6025 (3964 , 8709)	27.3 (18 , 39)	9.2 (-24.8 , 58.6)
Algeria	805 (630 , 1000)	2.1 (1.7 , 2.6)	69.1 (23.8 , 127)	291 (224 , 361)	0.9 (0.7 , 1.1)	24.1 (-8.7 , 64.7)	8712 (6842 , 10,870)	23.4 (18.3 , 29.1)	22.9 (-9.2 , 65.2)
Bahrain	55 (43 , 71)	4.6 (3.6 , 5.7)	32.3 (-1.8 , 73.1)	19 (14 , 24)	2.2 (1.7 , 2.7)	-7.3 (-32 , 24.2)	582 (438 , 741)	48.8 (37.7 , 61.4)	-11.6 (-35.3 , 18.4)
Egypt	1727 (1227 , 2385)	2.2 (1.6 , 3.1)	109.9 (45.5 , 198.8)	678 (475 , 983)	1 (0.7 , 1.5)	70.9 (18.2 , 151.7)	22,790 (16,244 , 31,572)	29.3 (20.7 , 41.4)	53.3 (7.3 , 121.7)
Iran	2579 (2351 , 2801)	3.3 (3 , 3.5)	81.5 (41.1 , 152.4)	934 (837 , 1018)	1.3 (1.2 , 1.5)	39.8 (12.8 , 121.7)	26,586 (24,228 , 28,540)	34.4 (31.1 , 37)	28.1 (0.3 , 83.9)
Iraq	1137 (857 , 1460)	4 (3 , 5)	93.1 (24.7 , 220.4)	428 (324 , 541)	1.8 (1.4 , 2.3)	48.1 (-4.8 , 156.7)	13,886 (10,454 , 17,719)	49.2 (37.1 , 62.5)	39.9 (-12 , 138.5)
Jordan	251 (203 , 306)	3.1 (2.5 , 3.7)	129.7 (64.5 , 206.9)	86 (69 , 105)	1.4 (1.1 , 1.7)	72.7 (23.8 , 144.9)	2691 (2167 , 3274)	34.2 (27.6 , 41.5)	64.2 (15.7 , 122)
Kuwait	130 (105 , 161)	4 (3.2 , 4.9)	32.4 (2.7 , 69.9)	37 (29 , 46)	1.5 (1.2 , 1.9)	21.8 (-2.5 , 52.8)	1159 (921 , 1454)	37.6 (29.8 , 47.2)	3.7 (-17.8 , 31.8)
Lebanon	287 (217 , 382)	5.5 (4.1 , 7.4)	162.6 (80.9 , 304.6)	125 (93 , 170)	2.4 (1.8 , 3.3)	80 (21.4 , 181.9)	2937 (2181 , 3979)	56.6 (42.1 , 76.9)	72 (16.3 , 167.7)
Libya	231 (149 , 308)	4.2 (2.7 , 5.6)	74.4 (-15 , 222.5)	109 (66 , 147)	2.2 (1.3 , 3)	42.6 (-34.7 , 177.8)	3044 (1864 , 4135)	55.4 (33.9 , 75.4)	39.6 (-37.4 , 169.7)
Morocco	573 (420 , 739)	1.7 (1.3 , 2.2)	113.1 (53.1 , 197.3)	264 (195 , 334)	0.9 (0.6 , 1.1)	73.4 (23.7 , 142.6)	7645 (5572 , 9796)	22.7 (16.6 , 28.8)	60 (14.7 , 120.9)
Oman	79 (60 , 94)	3.2 (2.6 , 3.8)	165.7 (71.7 , 311.8)	22 (17 , 27)	1.4 (1.1 , 1.6)	101.6 (28.8 , 239.3)	765 (583 , 940)	33 (26 , 38.7)	80.3 (11.9 , 188.2)
Palestine	99 (83 , 118)	3.3 (2.7 , 3.9)	64.3 (14.5 , 192.3)	37 (31 , 45)	1.6 (1.3 , 1.9)	41.8 (-3.6 , 178)	1205 (1014 , 1425)	40.3 (33.8 , 48.1)	35.3 (-7.4 , 149.3)
Qatar	69 (49 , 95)	6.9 (4.9 , 9.4)	77.1 (9.3 , 205)	22 (15 , 31)	4.1 (2.8 , 5.6)	46.4 (-10.7 , 160.6)	713 (492 , 1009)	72.8 (50 , 100.2)	17.2 (-29.8 , 107.6)
Saudi Arabia	1002 (746 , 1358)	4 (3.1 , 5.1)	237.3 (123.7 , 513.6)	287 (216 , 382)	1.6 (1.3 , 2)	124.4 (43.1 , 332.9)	10,001 (7407 , 13,450)	40.6 (31.4 , 52.3)	118.9 (41 , 299.5)
Sudan	594 (360 , 930)	2.2 (1.3 , 3.5)	143 (54.1 , 238.3)	243 (131 , 407)	1.2 (0.6 , 2)	107.4 (31 , 184.1)	8989 (5049 , 14,957)	33.4 (18.2 , 56.4)	83.2 (11.5 , 161.1)
Syrian Arab Republic	209 (151 , 284)	1.5 (1.1 , 2.1)	98.3 (28.7 , 207.3)	79 (55 , 108)	0.7 (0.5 , 0.9)	45 (-6.9 , 138)	2402 (1686 , 3282)	17.7 (12.5 , 24)	40 (-12.4 , 122.9)
Tunisia	351 (247 , 485)	2.8 (2 , 3.8)	103.8 (37.6 , 200.9)	146 (102 , 201)	1.2 (0.8 , 1.6)	53.1 (3.3 , 140.3)	3717 (2602 , 5116)	29.3 (20.6 , 40)	46.9 (-0.4 , 123.5)
Turkey	4349 (3421 , 5429)	5 (4 , 6.2)	77.4 (29.7 , 142.5)	1701 (1353 , 2127)	2 (1.6 , 2.4)	18.9 (-15.8 , 71.3)	46,255 (36,445 , 58,274)	52.8 (41.8 , 65.8)	8.8 (-23.2 , 52.4)
United Arab Emirates	621 (269 , 1051)	8.4 (3.4 , 13.6)	98.5 (-7.6 , 245.3)	238 (92 , 393)	4.7 (1.6 , 7.6)	68.9 (-22.3 , 198.8)	9420 (3795 , 15,697)	123.7 (47.1 , 197.5)	63.9 (-26.9 , 198.7)
Yemen	265 (177 , 386)	1.4 (0.9 , 1.9)	74.9 (11.8 , 184.6)	107 (70 , 152)	0.8 (0.5 , 1.1)	44 (-8.9 , 148.9)	3919 (2558 , 5616)	20.4 (13.4 , 29.3)	36.2 (-16.7 , 125.7)

DALY = disability-adjusted-life-years. (Generated from data available from http://ghdx.healthdata.org/gbd-results-tool).

### Country level

**Figure 1:**
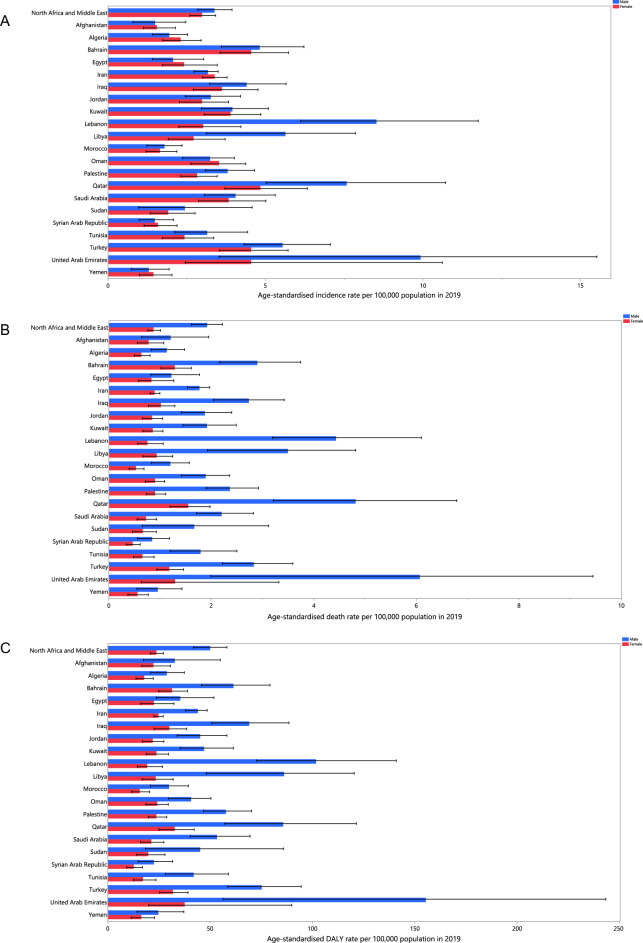
Age-standardised incidence (A), death (B), and DALYs (C) for kidney cancer (per 100,000 population) in the Middle East and North Africa region in 2019, by sex and country. DALY = disability-adjusted-life-years. (Generated from data available from http://ghdx.healthdata.org/gbd-results-tool).

At the country level, the age-standardised incidence rate of kidney cancer in 2019 exhibited significant variation, ranging from 1.4 to 8.4 cases per 100,000 individuals. The largest age-standardised incidence rate was observed in the United Arab Emirates, with 8.4 cases per 100,000 (3.4 to 13.6), followed by Qatar, with 6.9 cases per 100,000 (4.9 to 9.4), and Lebanon, with 5.5 (4.1 to 7.4). Conversely, the smallest rates were found in Yemen [1.4 (0.9 to 1.9)], Afghanistan [1.5 (1.0 to 2.1)], and the Syrian Arab Republic [1.5 (1.1 to 2.1)] (Table S2). Figure 1A provides a detailed visualization of the age-standardised incidence rates of the disease for each country in 2019, highlighting the considerable disparities across the region.

At the country level, the age-standardised mortality rate of kidney cancer varied from 0.7 to 4.7 cases per 100,000. The largest age-standardised mortality rates were observed in the United Arab Emirates [4.7 (1.6 to 7.6)], Qatar [4.1 (2.8 to 5.6)], and Lebanon [2.4 (1.8 to 3.3)], with the Syrian Arab Republic [0.7 (0.5 to 0.9)], Yemen [0.8 (0.5 to 1.1)], and Morocco [0.9 (0.6 to 1.1)] having the smallest (Table S3). Figure 1B shows the age-standardised mortality rates of kidney cancer by sex for each country in 2019.

In 2019, the age-standardised DALY rate of kidney cancer varied between 17.7 and 123.7 cases per 100,000 across the countries in the region. The largest rates were observed in the United Arab Emirates [123.7 (47.1 to 197.5)], Qatar [72.8 (50.0 to 100.2)], and Lebanon [56.6 (42.1 to 76.9)], while the lowest were in the Syrian Arab Republic [17.7 (12.5 to 24.0)], Yemen [20.4 (13.4 to 29.3)], and Morocco [22.7 (16.6 to 28.8)] (Table S4). Figure 1C shows the age-standardised DALY rates of kidney cancer for each sex in 2019.

The age-standardised incidence rate of kidney cancer increased in 17 of the MENA countries between 1990 and 2019, with no significant changes in the remainder. The largest increases were observed in Saudi Arabia [237.3% (123.7 to 513.6)], Oman [165.7% (71.7 to 311.8)], and Lebanon [162.6% (80.9 to 304.6)] (Fig. S1 and Table S2).

The age-standardised death rate of the disease was markedly higher in 2019 compared to 1990 in nine of the countries, with no significant changes in the rest. Saudi Arabia [124.4% (43.1 to 332.9)], Sudan [107.4% (31.0 to 184.1)], and Oman [101.6% (28.8 to 239.3)] experienced the most substantial increases in the age-standardised death rates (Fig. S2 and Table S3).

The age-standardised DALY rate of kidney cancer was higher in 2019 than in 1990 in eight of the countries, with no significant changes in the remainder. The largest increases were observed in Saudi Arabia [118.9% (41.0 to 299.5)], Sudan [83.2% (11.5 to 161.1)], and Oman [80.3% (11.9 to 188.2)] (Fig. S3 and Table S4).

### Age and sex patterns

**Figure 2:**
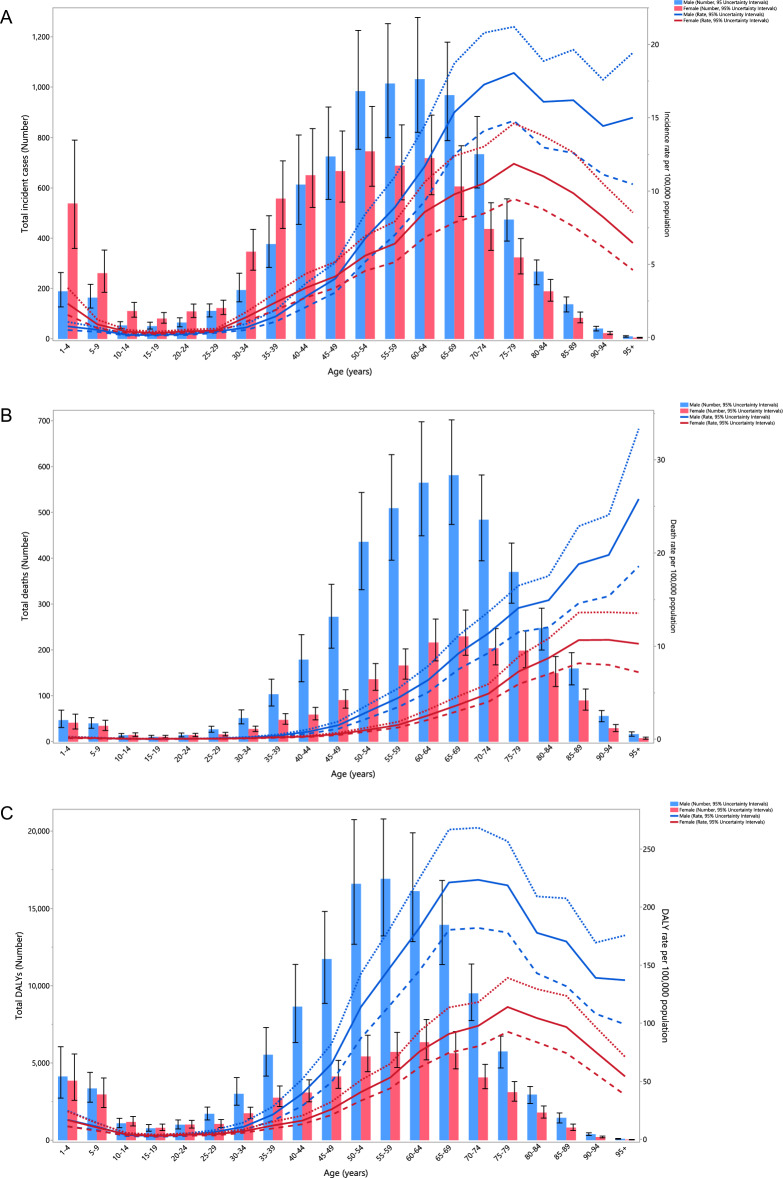
Number of incident cases and incidence rate (A), number of death cases and death rate (B), and the number of DALYs and DALY rate (C) for kidney cancer (per 100,000 population) in the Middle East and North Africa region, by age and sex in 2019; Dotted and dashed lines indicate 95% upper and lower uncertainty intervals, respectively. DALY = disability-adjusted-life-years. (Generated from data available from http://ghdx.healthdata.org/gbd-results-tool).

In 2019, the incidence rate for both sexes exhibited a notable decline with age up to those aged 10–14, remained stable up to the 25–29 age range, and then dramatically increased to the 75–79 age range, before decreasing up to those aged 95 + . The total number of incident cases of kidney cancer showed a similar pattern: it decreased with age up to the 15–19 age range for both males and females, then increased to the 60–64 age range and then declined to the 95 + age range. In 2019, the incident cases of the disease were higher in females up to the age of 44, after which the incidence rate was higher among men (Fig. 2A).

The mortality rate of kidney cancer in 2019 remained stable for both sexes in the 1–34 age range, and then rose with age. Furthermore, in 2019 the regional number of deaths due to kidney cancer decline up to the 15–19 age range, increased to the 65–69 age range, and then declined with age. Additionally, the number of deaths and the death rate were generally lower in women (Fig. 2B).

In 2019, the DALY rate of kidney cancer in the MENA region decreased up to the 10–14 age range for both sexes, remained unchanged up to the 25–29 age range, rose dramatically to the 65–69 age range and then declined to the 95 + age range. Also in 2019, the total number of DALYs in the MENA region decreased for both males and females up to the 15–19 age range, then increased up to the 55–59 age range and then declined to the 95 + age range. Overall, the number of DALYs and the DALY rate were again higher among males (Fig. 2C).

**Figure 3:**
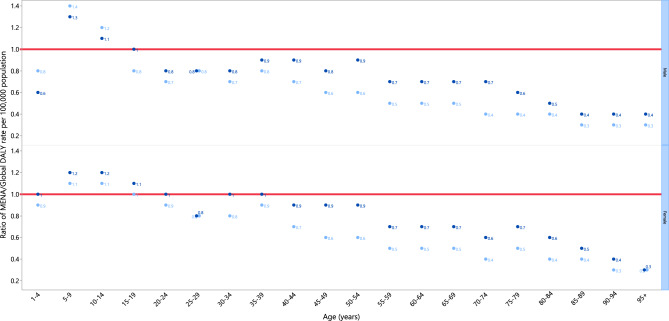
Ratio of the Middle East and North Africa region’s DALY rate to the global DALY rate of kidney cancer by age group and sex, 1990–2019. DALY = disability-adjusted-life-years. (Generated from data available from http://ghdx.healthdata.org/gbd-results-tool).

The DALY rate in 2019 was below the global DALY rate (ratio of MENA/Global DALY rate < 1) for males of most ages, except for those in the 5–19 age range. Specifically, the DALY rate in MENA was higher for males in the 5–14 age range, but was similar for those aged 15–19. For females, the MENA/Global DALY ratio in 2019 was higher among those aged 5–19, similar in the 1–4, 20–24, and 30–39 age ranges and lower in the other age ranges. When comparing 1990 to 2019, the MENA/Global DALY ratios for males were larger for all ages, except for the 1–19 range (lower) and the 25–29 range (similar). For females, the MENA/Global DALY ratios increased in all age groups between 1990 and 2019, except for the 25–29 and the 95 + age ranges, which were similar (Fig. 3).

### Relationship with the Socio-demographic Index (SDI)

**Figure 4:**
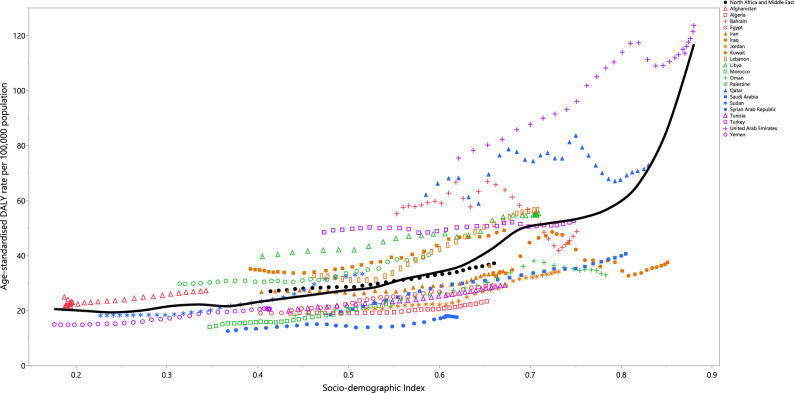
Age-standardised DALY rates of kidney cancer for the 21 MENA countries in 2019, by SDI; Expected values based on the Socio-demographic Index and disease rates in all locations are shown as the black line. Each point shows the observed age-standardised DALY rate for each country in 2019. DALY = disability-adjusted-life-years. SDI = Socio-demographic Index (Generated from data available from http://ghdx.healthdata.org/gbd-results-tool).

From 1990 to 2019, the age-standardised DALY rate of kidney cancer showed a consistent upward trend with increases in the SDI. Notably, several countries experienced a larger than expected age-standardised DALY rate, including the United Arab Emirates, Qatar, Afghanistan, Palestine, Iraq, Libya, and Lebanon. Conversely, Morocco, Tunisia, Kuwait, Yemen, Jordan, Oman, Algeria, Saudi Arabia, Egypt, and the Syrian Arab Republic were lower than anticipated (Fig. 4).

## Discussion

Our findings revealed that in 2019, the age-standardised incidence, death and DALY rates for kidney cancer were 3.2, 1.4 and 37.2, respectively. Additionally, these rates all significantly increased from 1990 to 2019. The data also indicated that both mortality and morbidity were higher among men, as well as among middle-aged and elderly populations. Furthermore, there was a clear positive correlation between a nation’s level of socioeconomic development and its burden of kidney cancer. This suggests that as countries progress socioeconomically, the burden of kidney cancer also escalates, potentially due to factors such as lifestyle changes, increased life expectancy, and improved diagnostic capabilities.

In 2017, the global age‑standardized rates for kidney cancer were 4.9 per 100,000 for incidence (95% UI: 4.7–5.1), 1.7 per 100,000 for mortality (95% UI: 1.6–1.8) and 41.1 per 100,000 for DALYs (95% UI: 38.7–42.5)^8^. In contrast, the present study reported corresponding figures of 3.2, 1.4 and 37.2, respectively. These findings indicate that the incidence and mortality rates in the region were below than the global average, although the DALY rate showed no significant difference. Socioeconomic factors contribute to the rarity of early detection and treatment in the MENA region, hindering the prevention of disease progression and complications. In 2017, the incidence rate of kidney cancer in this region was notably higher among men compared to women^8^, but in 2019 this gender disparity had diminished. The highest number of deaths occurred among those aged 65–69, with an overall increase in mortality across all age ranges. Consistent with global trends, the death rate remained higher among males than females in the MENA region, as was also the case in 2017^8^.

Previous studies have indicated that as countries become more developed, the incidence of kidney cancer increases. The highest incidence rates have been observed in North America and Eastern Europe, with European countries also exhibiting the highest death rates^8,14^. The current research demonstrated that the level of sociodemographic development plays a substantial role in the age-standardized DALY rate of kidney cancer in the population. The countries with high SDI levels (i.e., Kuwait, Bahrain, Saudi Arabia, and the United Arab Emirates) all had above average burdens. Although the overall burden of kidney cancer tends to rise with higher SDI levels, the relationship is not strictly linear. A global study also reported a slight increase up to an SDI of about 0.3, followed by a larger rise that peaked at its highest slope between 0.5 and 0.7, before decreasing again^8^. There are several plausible reasons why higher SDI countries experience a greater burden of kidney cancer. One significant factor is the utilisation of advanced diagnostic techniques, which include magnetic resonance imaging, ultrasonography, and computed tomography. These technologies, predominantly available in high SDI and high-income countries, can lead to overdiagnosis. This increased detection capability may contribute to the higher reported incidence of kidney cancer in these regions^15^.

Previous studies have also identified hypertension as a potent risk factor for kidney cancer^16,17^. In a 2021 study by Mills et al., it was found that the prevalence of hypertension in MENA was less than 28% among men, marking the lowest prevalence rate worldwide^18^. In addition, its prevalence in women was 28–29.9%, which was also low compared to other regions in the world^18^. It should be noted that compared to 1975, systolic blood pressure has increased by 0–2 mmHg on average for men and decreased by more than 2 mmHg for women, which may be associated with the lower deaths and DALYs found in women^18^. The research by Mills et al., also found a global increase in the absolute burden of hypertension for both sexes between 2000 and 2010, except in MENA where the absolute burden in women slightly decreased^19^. Therefore, the lower incidence of kidney cancer in the MENA region, especially among women, may be attributable to the lower prevalence of hypertension in the region. However, it may be that the poor diagnostic policies and limited facilities in the MENA region has led to underestimating the prevalence of both hypertension and kidney cancer.

A high body mass index (BMI) and obesity are other risk factors for kidney cancer. A cohort study by Ga Eun Nam, involving 23.3 million East Asians, showed a positive correlation between BMI, waist circumference and the chances of developing kidney cancer^20^. This indicates that obesity could potentially be a risk factor for the disease^20^. In the 2016 report by the World Health Organization, they reported that obesity is more common in the MENA region than in other areas^21^. In countries such as the United Emirates, Jordan, Iraq, Egypt, Turkey, Saudi Arabia, and Libya the prevalence of obesity has been estimated to exceed 30%^21,22^. This suggests that the region has a higher prevalence of obesity than many other regions. Therefore, the relatively low rate of kidney cancer observed in the present study appears to be inconsistent with the high prevalence of obesity in this region.

Smoking is a predisposing factor for RCC^23,24^. Thus, we can expect a higher rate of kidney cancer in regions with higher smoking. In 2020, the global prevalence of smoking was estimated to be 32.6% for males and 6.5% females, resulting in 7.0 million deaths^25^. Furthermore, between 1990 and 2020 there was a 27.2% decrease in the prevalence of smoking among men and a 37.9% decrease among females^25^. This decline was larger among high-income nations, than it was in low-income countries^25^. More specifically, high income countries saw a decline of over 40% in smoking rates, while the decline was significantly smaller in most low- and middle-income countries^25^. Consequently, the higher incidences of kidney cancer in countries with higher SDI levels do not align well with the larger declines in smoking prevalence observed in these high SDI countries. In addition, the global decline in smoking and the global increase in kidney cancer are incompatible with the fact that smoking substantially contributes to kidney cancer. Furthermore, kidney cancer is more common in men who smoke more than among women. These statistics cannot rule out the adverse effect of smoking on kidney health, but they appear to attenuate the importance of smoking's impact, when compared to other risk factors. In contrast, from 1990 until the present the MENA region has had a lower prevalence of smoking than globally, so a part of lower kidney cancer rate may be attributable to this fact^25^.

There is accumulating evidence to suggest several new risk factors (e.g., alcohol consumption, low physical activity, and high parity among women) could play a role in kidney cancer^17^. At present, the available evidence is not sufficient to prove the abovementioned factors are causally related to kidney cancer^17^. Males are considerably more at risk of developing kidney cancer and tend to experience poorer outcomes compared to women. Differences in the occupational risk factors, genetics-based variations, sex hormones, and tumor characteristics all contribute to this finding. However, further research is needed in whether sex is an independent prognostic factor for kidney cancer risk^26^. A Swedish case–control study revealed that multi-parity increases the RCC cancer risk by up to 1.42 times, compared to nulliparous females^27^. Furthermore, in the Canadian cohort study parous females were found to be 1.78 times higher risk of having kidney cancer, when compared to nulliparous women^26^. Physiological and hormonal changes during pregnancy result in elevated estrogen levels, increased renal hyperfiltration, and weight gain. Each of these factors independently contributes to the risk of kidney cancer, thereby explaining the observed correlation between parity and the incidence of the disease^26^. The low risk of kidney cancer in females, which increases following pregnancy, would appear to indicate that sex hormones are involved, but this needs further investigation.

Alcohol consumption differs worldwide, ranging from the lowest consumption in MENA (< 1 L per capita annually) to the highest consumption in Central and Eastern Europe (> 12 L per capita)^28^. The global consumption of alcohol has risen from 5.9 L per capita in 1990 to 6.5 L in 2017^28^, which may be associated with renal cancers^29,30^, but further investigation into this issue is needed.

### Strengths and limitations

This research offers the most current data regarding the burden of kidney cancer in MENA, providing valuable insights for health policymakers. However, several limitations must be acknowledged when reading these results. Firstly, there is a possibility that the burden of kidney cancer in MENA may have been underestimated. This potential underestimation is due to sparse data and the fact that there are no comprehensive cancer registries in most of the MENA countries, especially in the low- or middle-income nations. Secondly, the study did not differentiate the burden of kidney cancer by histological type or by race/ethnicity, despite the significant sociocultural variation in this region. This lack of granularity may obscure important variations in the disease’s impact across different population groups. Thirdly, the study did not take into account the burden caused by each of the different risk factors. Future GBD studies should consider including this information to provide a more comprehensive understanding of the disease’s determinants. Addressing these limitations in future research will be crucial for developing targeted interventions and improving kidney cancer outcomes in MENA.

## Conclusions

There burden of kidney cancer in the region has surged significantly from 1990 to 2019. Despite this significant rise, the incidence and death rates remain below the global average. This growing burden necessitates urgent attention and focused efforts to enhance the early identification and treatment strategies for kidney cancer within the MENA region. Addressing this issue is crucial to mitigate the impact of kidney cancer on the population and improve overall health outcomes.

### Supplementary Information

Supplementary Figure S1.

Supplementary Figure S2.

Supplementary Figure S3.

Supplementary Table S1.

Supplementary Table S2.

Supplementary Table S3.

Supplementary Table S4.

Supplementary Legends.

## Data Availability

The data used for these analyses are all publicly available at http://ghdx.healthdata.org/gbd-results-tool.
