# Supplementary figures and images for: Kidney cancer in the Middle East and North Africa region: a 30-year analysis (1990–2019)
Source: Sci Rep. 2024 Jun 14;14:13710. doi: 10.1038/s41598-024-64521-7 (PMC11178886; doi:10.1038/s41598-024-64521-7)

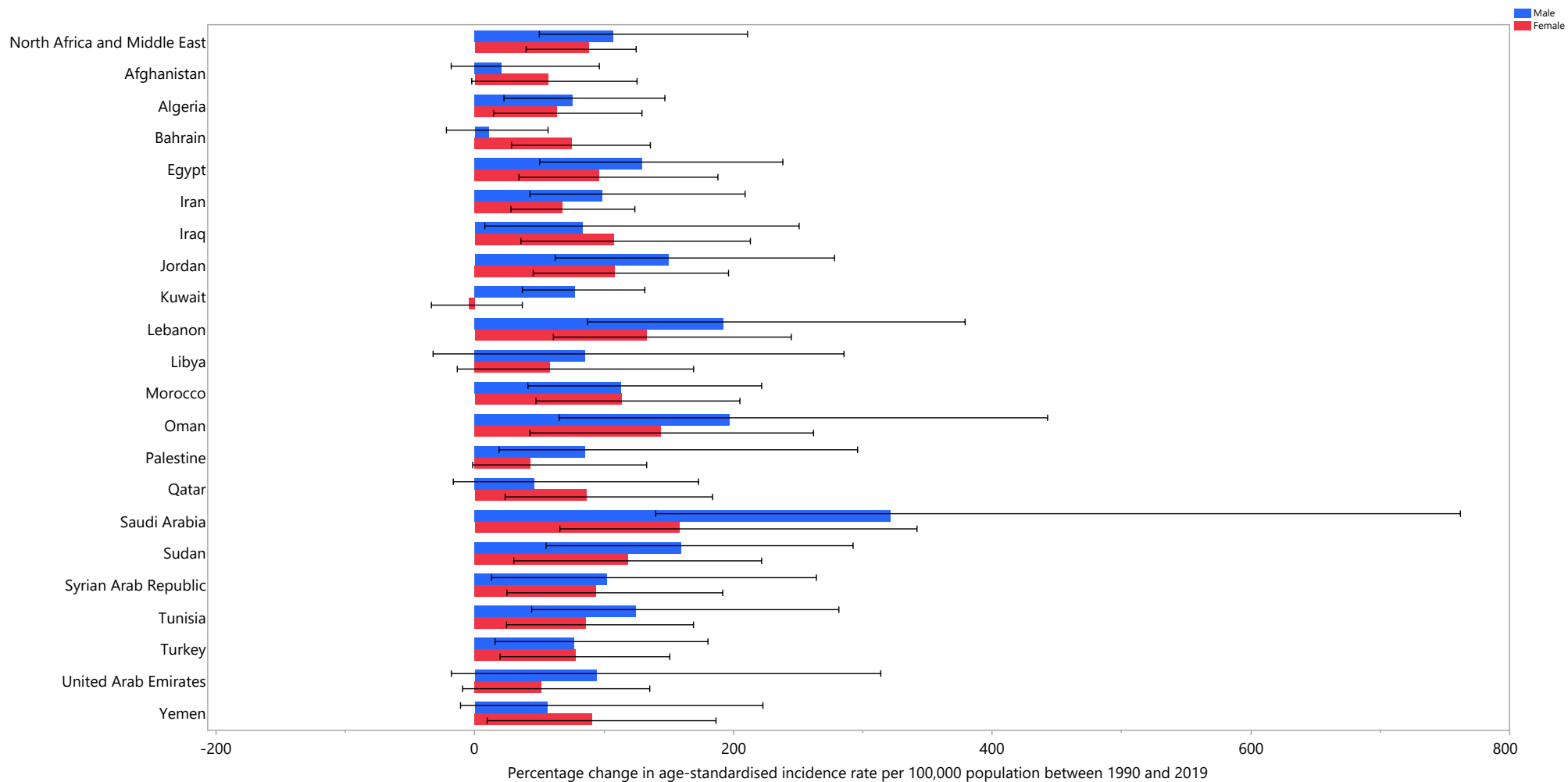

Supplement: Supplementary file 1 — Supplementary Figure S1. [file 41598_2024_64521_MOESM1_ESM.pdf]

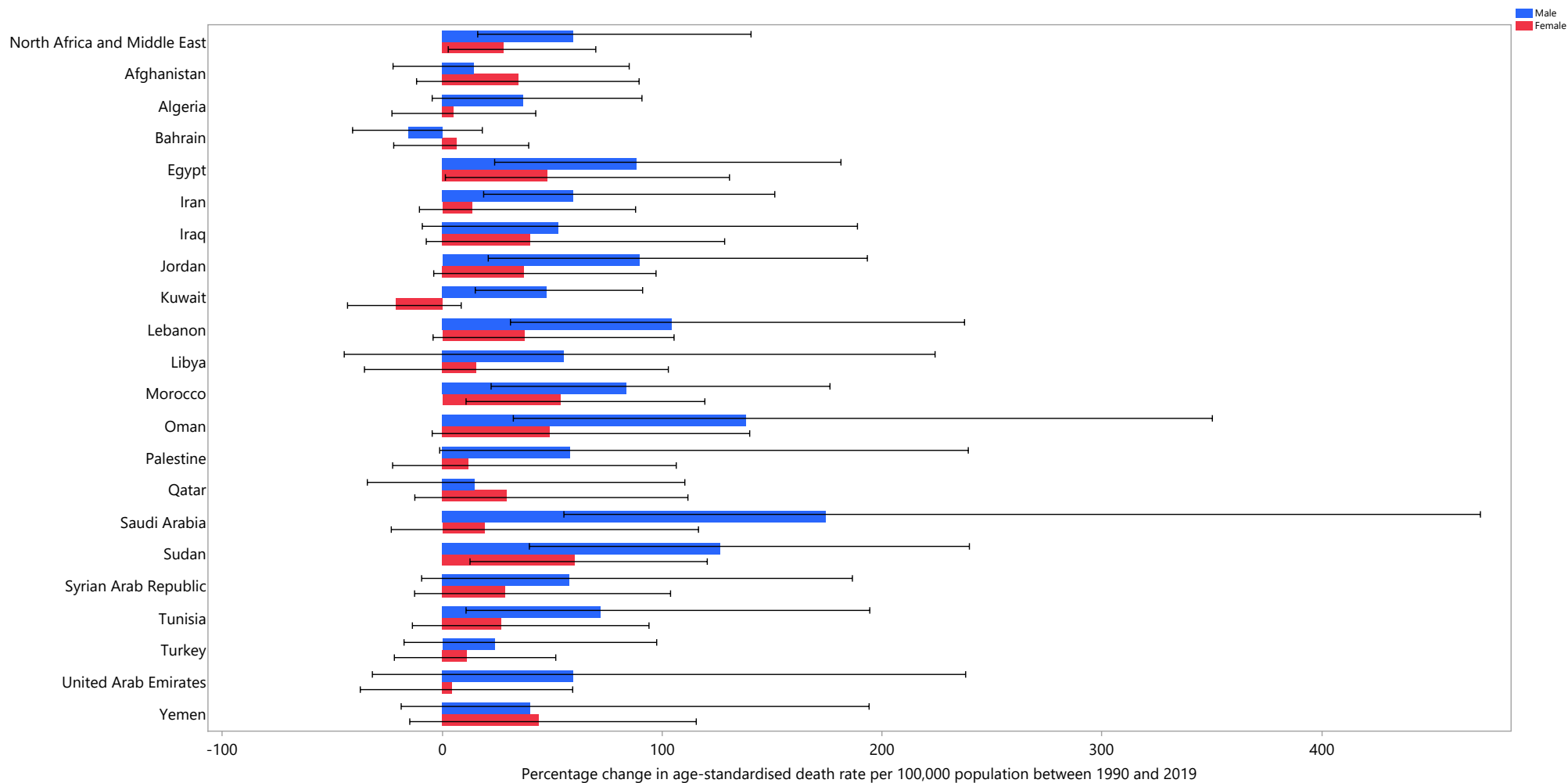

Supplement: Supplementary file 2 — Supplementary Figure S2. [file 41598_2024_64521_MOESM2_ESM.pdf]

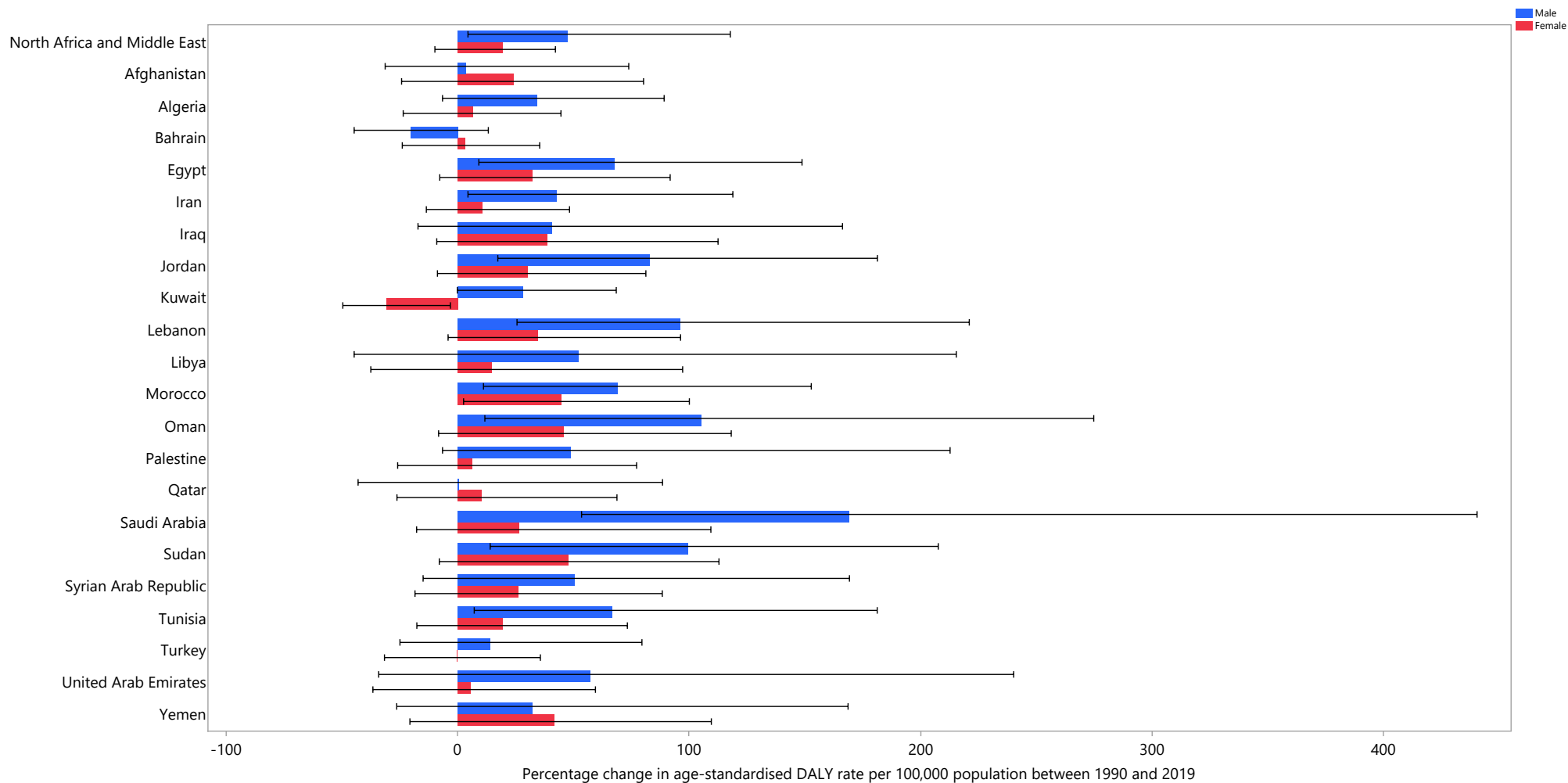

Supplement: Supplementary file 3 — Supplementary Figure S3. [file 41598_2024_64521_MOESM3_ESM.pdf]
