# Supplementary material for: Kidney cancer in the Middle East and North Africa region: a 30-year analysis (1990–2019)
Source: Sci Rep. 2024 Jun 14;14:13710. doi: 10.1038/s41598-024-64521-7 (PMC11178886; doi:10.1038/s41598-024-64521-7)
Supplement: Supplementary file 4 — Supplementary Table S1. [file 41598_2024_64521_MOESM4_ESM.docx]

| **Table S1: Sequelae for kidney cancer and the associated disability weights from the Global Burden of Disease 2019 Study** | | | |
| --- | --- | --- | --- |
| **Sequela** | **Lay description** | **Disability weight**  **(95% CI)** |  |
| Diagnosis and primary therapy phase of kidney cancer | This person has pain, nausea, fatigue, weight loss and high anxiety. | 0.288  (0.193-0.399) |  |
| Metastatic phase of kidney cancer | This person has severe pain, extreme fatigue, weight loss and high anxiety. | 0.451  (0.307-0.6) |  |
| Terminal phase of kidney cancer | This person has lost a lot of weight and regularly uses strong medication to avoid constant pain. The person has no appetite, feels nauseous, and needs to spend most of the day in bed. | 0.54  (0.377-0.687) |  |
| Controlled phase of kidney cancer | This person has a chronic disease that requires medication every day and causes some worry but minimal interference with daily activities. | 0.049  (0.031-0.072) |  |
