# Supplementary material for: Kidney cancer in the Middle East and North Africa region: a 30-year analysis (1990–2019)
Source: Sci Rep. 2024 Jun 14;14:13710. doi: 10.1038/s41598-024-64521-7 (PMC11178886; doi:10.1038/s41598-024-64521-7)
Supplement: Supplementary file 5 — Supplementary Table S2. [file 41598_2024_64521_MOESM5_ESM.docx]

| **Table S2: Incidence of kidney cancer in 1990 and 2019 and the percentage change in the age-standardised rates (ASRs) per 100,000 in the Middle East and North Africa region**  **(Generated from data available from http://ghdx.healthdata.org/gbd-results-tool)** | | | | | |
| --- | --- | --- | --- | --- | --- |
|  | **1990** | | **2019** | | **Percentage change in ASRs per 100,000** |
|  | **No (95% UI)** | **ASRs per 100,000 (95% UI)** | **No (95% UI)** | **ASRs per 100,000 (95% UI)** |  |
| **North Africa and Middle East** | **3873 (3310 , 4996)** | **1.6 (1.3 , 2)** | **15742 (13603 , 18040)** | **3.2 (2.8 , 3.6)** | **98 (55.5 , 157.4)** |
| **Afghanistan** | **105 (64 , 170)** | **1.1 (0.7 , 1.7)** | **316 (218 , 432)** | **1.5 (1 , 2.1)** | **33.8 (-6.7 , 92.9)** |
| **Algeria** | **207 (168 , 254)** | **1.3 (1 , 1.5)** | **805 (630 , 1000)** | **2.1 (1.7 , 2.6)** | **69.1 (23.8 , 127)** |
| **Bahrain** | **8 (6 , 9)** | **3.4 (2.9 , 4.1)** | **55 (43 , 71)** | **4.6 (3.6 , 5.7)** | **32.3 (-1.8 , 73.1)** |
| **Egypt** | **460 (397 , 547)** | **1.1 (1 , 1.2)** | **1727 (1227 , 2385)** | **2.2 (1.6 , 3.1)** | **109.9 (45.5 , 198.8)** |
| **Iran** | **757 (611 , 987)** | **1.8 (1.3 , 2.2)** | **2579 (2351 , 2801)** | **3.3 (3 , 3.5)** | **81.5 (41.1 , 152.4)** |
| **Iraq** | **227 (165 , 332)** | **2.1 (1.4 , 2.9)** | **1137 (857 , 1460)** | **4 (3 , 5)** | **93.1 (24.7 , 220.4)** |
| **Jordan** | **28 (23 , 35)** | **1.4 (1.1 , 1.7)** | **251 (203 , 306)** | **3.1 (2.5 , 3.7)** | **129.7 (64.5 , 206.9)** |
| **Kuwait** | **35 (29 , 43)** | **3 (2.6 , 3.5)** | **130 (105 , 161)** | **4 (3.2 , 4.9)** | **32.4 (2.7 , 69.9)** |
| **Lebanon** | **52 (41 , 67)** | **2.1 (1.7 , 2.7)** | **287 (217 , 382)** | **5.5 (4.1 , 7.4)** | **162.6 (80.9 , 304.6)** |
| **Libya** | **55 (38 , 78)** | **2.4 (1.6 , 3.4)** | **231 (149 , 308)** | **4.2 (2.7 , 5.6)** | **74.4 (-15 , 222.5)** |
| **Morocco** | **145 (114 , 182)** | **0.8 (0.6 , 1)** | **573 (420 , 739)** | **1.7 (1.3 , 2.2)** | **113.1 (53.1 , 197.3)** |
| **Oman** | **13 (9 , 17)** | **1.2 (0.8 , 1.6)** | **79 (60 , 94)** | **3.2 (2.6 , 3.8)** | **165.7 (71.7 , 311.8)** |
| **Palestine** | **26 (17 , 36)** | **2 (1.2 , 2.7)** | **99 (83 , 118)** | **3.3 (2.7 , 3.9)** | **64.3 (14.5 , 192.3)** |
| **Qatar** | **5 (4 , 7)** | **3.9 (2.7 , 5.2)** | **69 (49 , 95)** | **6.9 (4.9 , 9.4)** | **77.1 (9.3 , 205)** |
| **Saudi Arabia** | **98 (67 , 135)** | **1.2 (0.8 , 1.6)** | **1002 (746 , 1358)** | **4 (3.1 , 5.1)** | **237.3 (123.7 , 513.6)** |
| **Sudan** | **144 (88 , 216)** | **0.9 (0.6 , 1.3)** | **594 (360 , 930)** | **2.2 (1.3 , 3.5)** | **143 (54.1 , 238.3)** |
| **Syrian Arab Republic** | **61 (48 , 78)** | **0.8 (0.6 , 1)** | **209 (151 , 284)** | **1.5 (1.1 , 2.1)** | **98.3 (28.7 , 207.3)** |
| **Tunisia** | **83 (67 , 101)** | **1.4 (1.1 , 1.7)** | **351 (247 , 485)** | **2.8 (2 , 3.8)** | **103.8 (37.6 , 200.9)** |
| **Turkey** | **1257 (1019 , 1672)** | **2.8 (2.3 , 3.6)** | **4349 (3421 , 5429)** | **5 (4 , 6.2)** | **77.4 (29.7 , 142.5)** |
| **United Arab Emirates** | **36 (21 , 79)** | **4.3 (1.8 , 10.3)** | **621 (269 , 1051)** | **8.4 (3.4 , 13.6)** | **98.5 (-7.6 , 245.3)** |
| **Yemen** | **68 (41 , 103)** | **0.8 (0.4 , 1.1)** | **265 (177 , 386)** | **1.4 (0.9 , 1.9)** | **74.9 (11.8 , 184.6)** |
