# Supplementary material for: Kidney cancer in the Middle East and North Africa region: a 30-year analysis (1990–2019)
Source: Sci Rep. 2024 Jun 14;14:13710. doi: 10.1038/s41598-024-64521-7 (PMC11178886; doi:10.1038/s41598-024-64521-7)
Supplement: Supplementary file 6 — Supplementary Table S3. [file 41598_2024_64521_MOESM6_ESM.docx]

| **Table S3: Deaths from kidney cancer in 1990 and 2019 and the percentage change in the age-standardised rates (ASRs) per 100,000 in the Middle East and North Africa region**  **(Generated from data available from http://ghdx.healthdata.org/gbd-results-tool)** | | | | | |
| --- | --- | --- | --- | --- | --- |
|  | **1990** | | **2019** | | **Percentage change in ASRs per 100,000** |
|  | **No (95% UI)** | **ASRs per 100,000 (95% UI)** | **No (95% UI)** | **ASRs per 100,000 (95% UI)** |  |
| **North Africa and Middle East** | **1771 (1418 , 2147)** | **0.9 (0.7 , 1.1)** | **6003 (5161 , 6891)** | **1.4 (1.2 , 1.6)** | **48.9 (16.8 , 109.1)** |
| **Afghanistan** | **66 (38 , 105)** | **0.8 (0.5 , 1.3)** | **143 (94 , 205)** | **1 (0.6 , 1.4)** | **18.8 (-16.4 , 69.4)** |
| **Algeria** | **89 (72 , 108)** | **0.7 (0.6 , 0.9)** | **291 (224 , 361)** | **0.9 (0.7 , 1.1)** | **24.1 (-8.7 , 64.7)** |
| **Bahrain** | **4 (3 , 5)** | **2.3 (1.9 , 2.8)** | **19 (14 , 24)** | **2.2 (1.7 , 2.7)** | **-7.3 (-32 , 24.2)** |
| **Egypt** | **205 (186 , 227)** | **0.6 (0.6 , 0.7)** | **678 (475 , 983)** | **1 (0.7 , 1.5)** | **70.9 (18.2 , 151.7)** |
| **Iran** | **274 (197 , 328)** | **1 (0.6 , 1.1)** | **934 (837 , 1018)** | **1.3 (1.2 , 1.5)** | **39.8 (12.8 , 121.7)** |
| **Iraq** | **107 (72 , 161)** | **1.2 (0.8 , 1.8)** | **428 (324 , 541)** | **1.8 (1.4 , 2.3)** | **48.1 (-4.8 , 156.7)** |
| **Jordan** | **11 (9 , 14)** | **0.8 (0.6 , 1)** | **86 (69 , 105)** | **1.4 (1.1 , 1.7)** | **72.7 (23.8 , 144.9)** |
| **Kuwait** | **9 (8 , 10)** | **1.2 (1 , 1.4)** | **37 (29 , 46)** | **1.5 (1.2 , 1.9)** | **21.8 (-2.5 , 52.8)** |
| **Lebanon** | **30 (23 , 38)** | **1.3 (1.1 , 1.7)** | **125 (93 , 170)** | **2.4 (1.8 , 3.3)** | **80 (21.4 , 181.9)** |
| **Libya** | **30 (19 , 43)** | **1.6 (1 , 2.2)** | **109 (66 , 147)** | **2.2 (1.3 , 3)** | **42.6 (-34.7 , 177.8)** |
| **Morocco** | **73 (57 , 89)** | **0.5 (0.4 , 0.6)** | **264 (195 , 334)** | **0.9 (0.6 , 1.1)** | **73.4 (23.7 , 142.6)** |
| **Oman** | **5 (3 , 7)** | **0.7 (0.5 , 0.9)** | **22 (17 , 27)** | **1.4 (1.1 , 1.6)** | **101.6 (28.8 , 239.3)** |
| **Palestine** | **11 (6 , 15)** | **1.1 (0.7 , 1.6)** | **37 (31 , 45)** | **1.6 (1.3 , 1.9)** | **41.8 (-3.6 , 178)** |
| **Qatar** | **3 (2 , 4)** | **2.8 (1.8 , 3.7)** | **22 (15 , 31)** | **4.1 (2.8 , 5.6)** | **46.4 (-10.7 , 160.6)** |
| **Saudi Arabia** | **45 (28 , 61)** | **0.7 (0.4 , 1)** | **287 (216 , 382)** | **1.6 (1.3 , 2)** | **124.4 (43.1 , 332.9)** |
| **Sudan** | **68 (42 , 107)** | **0.6 (0.3 , 0.9)** | **243 (131 , 407)** | **1.2 (0.6 , 2)** | **107.4 (31 , 184.1)** |
| **Syrian Arab Republic** | **27 (20 , 34)** | **0.5 (0.3 , 0.6)** | **79 (55 , 108)** | **0.7 (0.5 , 0.9)** | **45 (-6.9 , 138)** |
| **Tunisia** | **39 (30 , 49)** | **0.8 (0.6 , 1)** | **146 (102 , 201)** | **1.2 (0.8 , 1.6)** | **53.1 (3.3 , 140.3)** |
| **Turkey** | **629 (489 , 807)** | **1.6 (1.3 , 2.1)** | **1701 (1353 , 2127)** | **2 (1.6 , 2.4)** | **18.9 (-15.8 , 71.3)** |
| **United Arab Emirates** | **16 (7 , 36)** | **2.8 (1 , 7.1)** | **238 (92 , 393)** | **4.7 (1.6 , 7.6)** | **68.9 (-22.3 , 198.8)** |
| **Yemen** | **32 (18 , 47)** | **0.5 (0.3 , 0.8)** | **107 (70 , 152)** | **0.8 (0.5 , 1.1)** | **44 (-8.9 , 148.9)** |
