# Supplementary material for: Kidney cancer in the Middle East and North Africa region: a 30-year analysis (1990–2019)
Source: Sci Rep. 2024 Jun 14;14:13710. doi: 10.1038/s41598-024-64521-7 (PMC11178886; doi:10.1038/s41598-024-64521-7)
Supplement: Supplementary file 7 — Supplementary Table S4. [file 41598_2024_64521_MOESM7_ESM.docx]

| **Table S4: DALYs due to kidney cancer in 1990 and 2019 and the percentage change in the age-standardised rates (ASRs) per 100,000 in the Middle East and North Africa region**  **(Generated from data available from http://ghdx.healthdata.org/gbd-results-tool)** | | | | | |
| --- | --- | --- | --- | --- | --- |
|  | **1990** | | **2019** | | **Percentage change in ASRs per 100,000** |
|  | **No (95% UI)** | **ASRs per 100,000 (95% UI)** | **No (95% UI)** | **ASRs per 100,000 (95% UI)** |  |
| **North Africa and Middle East** | **66722 (55206 , 84585)** | **27 (21.8 , 33.1)** | **183630 (157587 , 211815)** | **37.2 (32 , 42.6)** | **37.7 (5.8 , 83.6)** |
| **Afghanistan** | **2550 (1433 , 4284)** | **25 (14.4 , 40.3)** | **6025 (3964 , 8709)** | **27.3 (18 , 39)** | **9.2 (-24.8 , 58.6)** |
| **Algeria** | **3162 (2593 , 3861)** | **19 (15.4 , 23.2)** | **8712 (6842 , 10870)** | **23.4 (18.3 , 29.1)** | **22.9 (-9.2 , 65.2)** |
| **Bahrain** | **124 (102 , 151)** | **55.2 (44.9 , 68.1)** | **582 (438 , 741)** | **48.8 (37.7 , 61.4)** | **-11.6 (-35.3 , 18.4)** |
| **Egypt** | **8647 (7526 , 9993)** | **19.1 (17.3 , 21.3)** | **22790 (16244 , 31572)** | **29.3 (20.7 , 41.4)** | **53.3 (7.3 , 121.7)** |
| **Iran** | **11442 (8864 , 14583)** | **26.8 (19.5 , 32.2)** | **26586 (24228 , 28540)** | **34.4 (31.1 , 37)** | **28.1 (0.3 , 83.9)** |
| **Iraq** | **3957 (2779 , 5860)** | **35.2 (23.5 , 52.6)** | **13886 (10454 , 17719)** | **49.2 (37.1 , 62.5)** | **39.9 (-12 , 138.5)** |
| **Jordan** | **421 (349 , 530)** | **20.8 (16.7 , 26.1)** | **2691 (2167 , 3274)** | **34.2 (27.6 , 41.5)** | **64.2 (15.7 , 122)** |
| **Kuwait** | **398 (339 , 464)** | **36.2 (31 , 42)** | **1159 (921 , 1454)** | **37.6 (29.8 , 47.2)** | **3.7 (-17.8 , 31.8)** |
| **Lebanon** | **833 (646 , 1074)** | **32.9 (25.6 , 42.3)** | **2937 (2181 , 3979)** | **56.6 (42.1 , 76.9)** | **72 (16.3 , 167.7)** |
| **Libya** | **939 (618 , 1378)** | **39.7 (25.1 , 56.7)** | **3044 (1864 , 4135)** | **55.4 (33.9 , 75.4)** | **39.6 (-37.4 , 169.7)** |
| **Morocco** | **2567 (1991 , 3208)** | **14.2 (11.1 , 17.4)** | **7645 (5572 , 9796)** | **22.7 (16.6 , 28.8)** | **60 (14.7 , 120.9)** |
| **Oman** | **185 (122 , 255)** | **18.3 (11.9 , 25)** | **765 (583 , 940)** | **33 (26 , 38.7)** | **80.3 (11.9 , 188.2)** |
| **Palestine** | **384 (249 , 527)** | **29.8 (17.8 , 41.1)** | **1205 (1014 , 1425)** | **40.3 (33.8 , 48.1)** | **35.3 (-7.4 , 149.3)** |
| **Qatar** | **88 (62 , 120)** | **62.2 (41.3 , 83.9)** | **713 (492 , 1009)** | **72.8 (50 , 100.2)** | **17.2 (-29.8 , 107.6)** |
| **Saudi Arabia** | **1614 (1074 , 2233)** | **18.6 (11.9 , 25.5)** | **10001 (7407 , 13450)** | **40.6 (31.4 , 52.3)** | **118.9 (41 , 299.5)** |
| **Sudan** | **3117 (1759 , 5242)** | **18.2 (11.2 , 28.6)** | **8989 (5049 , 14957)** | **33.4 (18.2 , 56.4)** | **83.2 (11.5 , 161.1)** |
| **Syrian Arab Republic** | **1000 (774 , 1293)** | **12.6 (9.5 , 16)** | **2402 (1686 , 3282)** | **17.7 (12.5 , 24)** | **40 (-12.4 , 122.9)** |
| **Tunisia** | **1224 (971 , 1518)** | **19.9 (15.5 , 24.7)** | **3717 (2602 , 5116)** | **29.3 (20.6 , 40)** | **46.9 (-0.4 , 123.5)** |
| **Turkey** | **21954 (17059 , 28928)** | **48.5 (38.1 , 62.7)** | **46255 (36445 , 58274)** | **52.8 (41.8 , 65.8)** | **8.8 (-23.2 , 52.4)** |
| **United Arab Emirates** | **686 (364 , 1541)** | **75.5 (31.3 , 183.1)** | **9420 (3795 , 15697)** | **123.7 (47.1 , 197.5)** | **63.9 (-26.9 , 198.7)** |
| **Yemen** | **1387 (788 , 2149)** | **15 (8.1 , 22.4)** | **3919 (2558 , 5616)** | **20.4 (13.4 , 29.3)** | **36.2 (-16.7 , 125.7)** |
