# Supplementary material for: Kidney cancer in the Middle East and North Africa region: a 30-year analysis (1990–2019)
Source: Sci Rep. 2024 Jun 14;14:13710. doi: 10.1038/s41598-024-64521-7 (PMC11178886; doi:10.1038/s41598-024-64521-7)
Supplement: Supplementary file 8 — Supplementary Legends. [file 41598_2024_64521_MOESM8_ESM.docx]

**Table S1:** Sequelae for kidney cancer and their associated disability weights, from the Global Burden of Disease 2019 Study

**Table S2**: Incidence of kidney cancer in 1990 and 2019 for both sexes and the percentage change in the age-standardised rates (ASRs) per 100,000 in the Middle East and North Africa region (Generated from data available from <http://ghdx.healthdata.org/gbd-results-tool>).

**Table S3:** Deaths from kidney cancer in 1990 and 2019 for both sexes and the percentage change in the age-standardised rates (ASRs) per 100,000 in the Middle East and North Africa region (Generated from data available from <http://ghdx.healthdata.org/gbd-results-tool>).

**Table S4:** DALYs due to kidney cancer in 1990 and 2019 for both sexes and the percentage change in the age-standardised rates (ASRs) per 100,000 in the Middle East and North Africa region (Generated from data available from <http://ghdx.healthdata.org/gbd-results-tool>).

**Figure S1:** The percentage change in the age-standardised incidence rate of kidney cancer in the Middle East and North Africa region from 1990 to 2019, by sex and country. (Generated from data available from <http://ghdx.healthdata.org/gbd-results-tool>).

**Figure S2:** The percentage change in the age-standardised death rate of kidney cancer in the Middle East and North Africa region from 1990 to 2019, by sex and country. (Generated from data available from <http://ghdx.healthdata.org/gbd-results-tool>).

**Figure S3:** The percentage change in the age-standardised DALY rate of kidney cancer in the Middle East and North Africa region from 1990 to 2019, by sex and country. DALY= disability-adjusted-life-years. (Generated from data available from <http://ghdx.healthdata.org/gbd-results-tool>).
